# Comparison of Neutralizing Antibody Responses Elicited from Highly Diverse Polyvalent Heterotrimeric HIV-1 gp140 Cocktail Immunogens versus a Monovalent Counterpart in Rhesus Macaques

**DOI:** 10.1371/journal.pone.0114709

**Published:** 2014-12-09

**Authors:** Emma J. Bowles, Torben Schiffner, Maximillian Rosario, Gemma A. Needham, Meghna Ramaswamy, Joanna McGouran, Benedikt Kessler, Celia LaBranche, Andrew J. McMichael, David Montefiori, Quentin J. Sattentau, Tomáš Hanke, Guillaume B. E. Stewart-Jones

**Affiliations:** 1 MRC Human Immunology Unit, Weatherall Institute of Molecular Medicine, University of Oxford, The John Radcliffe Hospital, Oxford, United Kingdom; 2 Sir William Dunn School of Pathology, University of Oxford, Oxford, United Kingdom; 3 Division of Retrovirology, Centre for AIDS Reagents, National Institute of Biological Standards and Control, South Mimms, Potters Bar, Herts, United Kingdom; 4 Henry Wellcome Building for Molecular Physiology, Nuffield Department of Medicine, University of Oxford, Oxford, United Kingdom; 5 Division of Surgical Sciences, Duke University Medical Center, Durham, North Carolina, United States of America; 6 The Jenner Institute, University of Oxford, Old Road Campus Research Building, Oxford, United Kingdom; Simon Fraser University, Canada

## Abstract

Eliciting neutralizing antibodies capable of inactivating a broad spectrum of HIV-1 strains is a major goal of HIV-1 vaccine design. The challenge is that envelopes (Envs) of circulating viruses are almost certainly different from any Env used in a vaccine. A novel immunogen composed of a highly diverse set of gp140 Envs including subtypes A, B, C, D and F was developed to stimulate a more cross-neutralizing antibody response. Env heterotrimers composed of up to 54 different gp140s were produced with the aim of focusing the response to the conserved regions of Env while reducing the dominance of any individual hypervariable region. Heterotrimeric gp140 Envs of inter- and intra-subtype combinations were shown to bind CD4 and a panel of neutralizing monoclonal antibodies with similar affinity to monovalent UG37 gp140. Macaques immunized with six groups of heterotrimer mixtures showed slightly more potent neutralizing antibody responses in TZM-BL tier 1 and A3R5 tier 2 pseudovirus assays than macaques immunized with monovalent Env gp140, and exhibited a marginally greater focus on the CD4-binding site. Carbopol enhanced neutralization when used as an adjuvant instead of RIBI in combination with UG37 gp140. These data indicate that cross-subtype heterotrimeric gp140 Envs may elicit some improvement of the neutralizing antibody response in macaques compared to monovalent gp140 Env.

## Introduction

The development of an effective HIV-1 vaccine remains a major global health priority: recent figures from the World Health Organisation indicate that approximately 35 million people were living with HIV-1 at the end of 2013. Whilst the availability of antiretroviral drugs can prolong the lives of HIV-1-positive individuals in countries where they are readily available, a protective vaccine would be the most effective way of reducing viral transmission. HIV-1 has diversified in the human population for many decades, and due to the extraordinary genetic variety of subtypes now in circulation, a vaccine needs to induce immunity that targets a wide range of epitopes or targets motifs that are conserved across most viruses. It is also possible that an effective HIV-1 vaccine may need to stimulate humoral and cellular immune responses; the recent RV144 trial in Thailand supported the hypothesis that improved protection could be achieved by combining a T-cell-stimulating vaccine with an antibody stimulating vaccine [1], [2].

HIV-1 Env is an extensively glycosylated trimer of gp120-gp41 heterodimers that is critical for binding to the main CD4 receptor and coreceptors; it facilitates entry into target cells [3] and is the target of neutralizing and broadly neutralizing antibodies (NAbs and bNABs). To date, various compositions of soluble gp120 and gp140 immunogens have been tested in immunogenicity studies (reviewed in [4]) and whilst improved responses were observed when using gp140 rather than gp120 immunogens [5]–[7], the majority did not elicit Ab responses capable of potently neutralizing diverse HIV-1 strains. However, passive transfer of bNAbs can provide protection against vaginal, oral and rectal challenge with SHIV [8]–[12], and vaccine-induced NAbs can be similarly protective [13], [14]. Therefore, it is crucial to understand how bNAbs can be elicited by active immunization.

During chronic infection, potent and cross-reactive NAbs (broadly neutralizing antibodies; bNAbs) that are capable of neutralizing heterologous viruses of diverse subtypes develop in of 10–25% HIV-1 infected individuals [15]–[18]. Characterisation of these responses has shown many of these bNAbs target the conserved regions near the CD4 binding site (CD4bs) [19] and sites at the base of the V3 and V1/V2 loops [20], of which some bNAbs are glycan-dependent [21]–[23] and some target the membrane-proximal external region (MPER) [24], [25]. The development of bNAbs was shown to correlate with high plasma viremia and could result from chronic and evolving antigen exposure over a number of years that has allowed sufficient somatic hypermutation in the B-cell receptors (BCRs) and focuses the B-cell response to the conserved neutralisation sites on Env [17]. Some isolated monoclonal bNAbs contain up to 35% somatic hypermutation of the BCRs [26], suggesting that during the course of infection B-cell responses to Env adapt to a mutating antigen over time, which may drive their focus to the most conserved epitope motifs.

A vaccine strategy that aims to mimic the diverse antigenic exposure experienced during natural infection may generate NAbs of greater breadth and potency. A polyvalent vaccine, comprising a combination of multiple Env proteins, was shown to be better in this respect to monovalent Env in both rabbits and macaques [27]–[30]. Morner *et al*. achieved greater focusing of the immune response on conserved regions of Env by sequential immunization of macaques with gp120 core protein followed by trimer boosting [31]. Similarly, the administration of sequential rather than single or mixed patient-derived gp140 Env genes in rabbits showed a marginal improvement in breadth of neutralization [32]. Sellhorn *et al*. recently described a novel assembly of gp140 heterotrimers which had gp140 subunits from two different genetic sources and improved potency of NAb responses in rabbits as compared to homotrimeric equivalents [33].

In this study, we created high antigenic diversity with the gp140 immunogens by forming heterotrimers from a diverse set of HIV-1-infected patient-derived Env genes from subtypes A, B, C, D and F, and compared their immunogenicity with homotrimeric UG37 gp140 in rhesus macaques. Inter and intra-subtype heterotrimers readily assembled. For comparison between the heterotrimers and homotrimers of gp140 immunogens, RIBI Adjuvant System was used, which is an oil (squalene)-in-water emulsion. An additional group of macaques was immunised with the homotrimeric gp140 adjuvanted with carbopol, which is a polyanionic carbomer gel shown to induce strong specific antibody responses to HIV-1 Env in mice [34] and rabbits [35], [36]. Carbopol is currently used in animal vaccinations but its use in humans has not been reported. In the macaque immunizations reported here, carbopol was well tolerated and resulted in robust anti-HIV-1 Env responses. An improved targeting to the CD4bs was detected both with the heterotrimer immunogens and with carbopol as an adjuvant compared to the monovalent UG37 with RIBI control group. Furthermore, a NAb response of improved potency against tier 1 and tier 2 HIV-1 pseudoviruses was achieved with heterotrimer mixes compared to homotrimeric gp140.

## Materials and Methods

### 2.1. Ethics statement

Approval for the work with non-human primates was granted by the Home Office under the authority of a Project Licence (Licence number PPL 30–2424), granted under the UK’s Animal (Scientific Procedures) Act and with the endorsement of the Ethical Review Committee at the University of Oxford.

### 2.2. Amplification and sequencing of HIV-1 Env genes

HIV-1 gp140 Env genes were amplified from 84 patient serum samples (collected by the Centre for AIDS Reagents at the National Institute for Biological Standards and Control, UK; NIBSC) and sequenced as previously described [37]. Viral RNA was isolated from patient sera and amplified using Superscript III One-step RT-PCR with High Fidelity Platinum *Taq* (Invitrogen) using primers EnvA (fw) and Env3Rlong (rev) [35]. Where necessary, a further 25-cycle nested PCR reaction was performed using Advantage 2 Polymerase mix (Clontech) with internal primers Env_2Flong (fw) and Nef5 (rev) [35]. All primer details can be found in S1 Table.

PCR products were cloned into the pCR 4-TOPO vector from the TOPO TA Cloning Kit for Sequencing (Invitrogen) and used to transform One-shot TOP10 chemically competent *Escherichia coli*. Individual colonies were picked and shipped to Functional Biosciences, Inc, Madison, WI, USA for sequencing with an Applied Biosystems 3730xl Genetic Analyzer, as described previously [37].

### 2.3. Construction of gp140 Env expression plasmids

All Env expression plasmids were made using the pLEXm vector, which is designed for protein expression in mammalian cells [38]. The human tissue plasminogen activator leader sequence was used to replace the native HIV-1 gp140 leader, a modification which has been shown to increase gp140 expression [39]. The 3′ primer incorporated a *Xho*I site and a His_6_ tag sequence followed by a stop codon. PCR products were digested with *EcoR*I (New England Biolabs, Beverly, MA; NEB) and *Xho*I (NEB), gel purified (Qiagen, Venlo, The Netherlands, and ligated into the pLEXm plasmid. Ligation reactions were performed at room temperature (RT) for 1 hr using T4 DNA ligase (NEB) prior to transformation into DH5 alpha *Escherichia coli* cells (Invitrogen, Carlsbad, CA). Following DNA extraction, plasmids were sequence-verified.

Gp140-encoded regions of *env* were amplified, incorporating the full native gp120 and gp41 mature protein encoding regions with the furin site replaced with SEKS and ending with amino-acid position 668, located at the membrane proximal region of gp41, followed by a His_6_ tag. All PCR reactions were performed with KOD DNA Polymerase (Novagen, Madison, WI), according to the manufacturer’s instructions. Primer details can be found in S1 Table.

### 2.4. Small-scale gp140 Env expression screen

**Figure 1:**
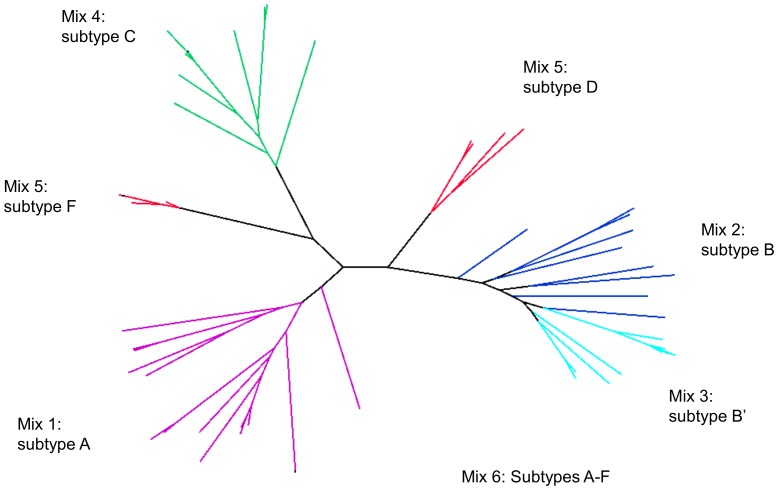
Genetic diversity of HIV-1 gp140s present in immunogen Mixes 1–6. The phylogeny was constructed from the *env* nucleotide sequence alignment using a maximum likelihood model. Edges are coloured according to mix. Due to a high representation of expressing subtype B Envs, the 19 subtype B Envs were partitioned into 2 subtype B groups designated B and B′.

Env plasmids were screened for protein expression using a 1 ml culture transfection protocol in high glucose DMEM media (Sigma, St. Louis, MO) supplemented with 10% fetal calf serum (FCS; Sigma) and Penicillin (100 units/ml) Streptomycin (10 ng/ml). Two µg of each DNA construct were incubated with 3.6 µg polyethylelimine (PEI; Sigma) in 150 µl DMEM media for 30 min. The DNA-PEI mixture was added to 90% confluent cells and the volume made up to 1 ml with DMEM containing 2% FCS. After 48–72 hr, supernatants were analyzed by gel electrophoresis on 4–12% Bis-Tris gels (Invitrogen) and western blot to detect protein expression levels. Blots were incubated overnight with a penta-His antibody (Qiagen) followed by 1 hr at RT with an anti-mouse secondary antibody conjugated with HRP (Invitrogen). A maximum likelihood phylogeny (TREE-PUZZLE [40]) was constructed from *envs* that were positive in the expression screen. Of these, expression plasmids were selected for inclusion in immunogen mixes based on their gp140 *env* sequence diversity; *envs* from individual subtypes were selected for the different mixes with the exception of Mix 5, which contained *envs* from both subtypes D and F due to the availability of nine unique genotypes available for these subtypes. Where possible, *envs* were selected from as many different patients as possible, in order to maximise the genetic diversity represented in each mix (Fig. 1).

### 2.5. Proof-of-concept of heterotrimeric gp140 production

In order to determine whether heterotrimers that contain three unique gp140 subunits could be produced, a two-stage capture experiment was designed. 293T cells were co-transfected with three *env* expression plasmids containing different C-terminal tags. Gp140 plasmids were cloned where the His_6_ tag was replaced by either FLAG: DYKDDDDK, HA: YPYDVPDYA, FLAG-His_6_: DYKDDDDKHHHHHH, or HA-His_6_: YPYDVPDYAHHHHHH tags. 72 hr following transfection, supernatants were centrifuged at 13,000 rpm for 5 mins, passed through a 22-µm filter, and adjusted to pH 8 using 1 M Tris-HCl, pH 8 (Sigma). This was passed over a 5 ml Talon metal-affinity Superflow Resin (Clontech) to specifically bind the His_6_ tag. Protein was eluted with 250 mM imidazole (Sigma) and the protein concentrated to 1 ml using 7 ml centrifugation columns for protein purification with 9,000 kDa molecular weight cut-off (Pierce, Rockford, IL). Protein was incubated with 20 µl anti-FLAG-tagged magnetic beads (Sigma) according to the manufacturer’s protocol, washed with 1 M Tris-HCl pH 8.0 and protein was eluted by competition with the FLAG peptide (Sigma). The proteins labelled with the different tags were detected by western blot, as described above; using different antibodies. FLAG expression was detected using an anti-FLAG M2 mouse monoclonal antibody (Invitrogen) and HA expression was detected using a mouse anti-HA antibody (AbCam, Cambridge, UK).

### 2.6. Expression and purification of gp140 Env immunogens

All gp140 Envs were produced by transient transfection in 293T cells cultured in multilayer Cell Bind Hyperflasks (Corning, NY each of which was transfected with 2 mg DNA. UG37 DNA was synthesised from the sequence available from GenBank accession number AAB05027.1. Cells at 80–90% confluency were transfected with gp140 DNA complexed with PEI at a ratio of 1 mg DNA: 1.8 mg PEI. DNA-PEI mixtures were incubated for 30 min prior to being added to the cells in DMEM with 2% FCS. Supernatants were collected after 48 hr and fresh media, this time containing 10% FCS, was added to the cells for a further 48 hr at which point the media were exchanged once again. All supernatants were centrifuged at 7000 x g for 4 hr to remove cell debris, and passed through a 0.22-µm filter. After adjusting to pH 8 using 1 M Tris-HCl (Sigma), media were passed over a Talon Superflow resin (Clontech, Mountain View, CA) column. After washing with 2 column volumes of 0.015 M Tris Buffered Saline (Sigma), protein was eluted with 250 mM imidazole. The eluted gp140 was concentrated and separated by size-exclusion chromatography (SEC) using a Superdex200 26/60 size-exclusion column (GE Healthcare, Pollards Wood, UK). Fractions corresponding to the trimer peak were identified and further purified using GNA-lectin (Vector Labs, Peterborough, UK) to specifically bind the glycoprotein. An additional SEC fractionation allowed separation of pure gp140.

### 2.7. SPR and ELISA Reagents

CD4-IgG2 [41] used in the binding studies was obtained through the NIH AIDS Reagent Program (source: Progenics Pharmaceuticals, Inc.), Division of AIDS, NIAID, NIH: Cat#11780. Soluble domains 1–4 CD4 (sCD4) and biotinylated domains 1–4 CD4 were a gift from Simon Davis [42]. 2G12 [43], 17b [44] and B6 [45] were obtained from the IAVI Neutralizing Antibody Consortium. 5F3 [43] was purchased from Polymun Scientific (Austria), VRC01 [46] was a gift from Dr Connors (VRC, NIAID). HGP68, HR10 and HJ16 [19] were a gift from D. Corti and A. Lanzavecchia (Institute for Research in Biomedicine, Switzerland). Biotinylated mAbs were produced using the EZ-Link NHS-LC-Biotin reagent (Fisher Scientific UK, Loughborough, UK) according to manufacturer’s instructions.

### 2.8. ELISA analysis of gp140-CD4 binding

Gp140s were coated on wells of 96-well ELISA plates at 2 µg/ml for 2 hr at 37°C. Plates were then washed three times with PBS containing 0.05% tween-20 (PBST) and blocked with blocking buffer (2% BSA in PBST) for 1 hr at RT. CD4-IgG2 was titrated onto the plates and incubated for 2 hr at RT followed by another three washes with PBST and a 1 hr incubation at RT with anti-human-IgG-HRP secondary antibody (Sigma) at a 1∶10,000 dilution in blocking buffer. Binding was detected with 1-Step Ultra TMB-ELISA Substrate (Thermo Scientific). The reaction was stopped by addition of 0.5 M H2SO4 and absorption was read at 450 nm. The absorbance of negative controls was subtracted from the data (∼0.1% of maximum binding values) and normalized data were fitted to a specific one-site binding model (R^2^ values of fit ranging from 0.93 to 0.99, mean 0.97, median 0.97) in GraphPad Prism v5.0 (GraphPad Software, Inc., San Diego, CA, USA). 50% effective concentrations (EC_50_) were defined as the concentration at which the fitted curve reached an OD of 50% of the maximum binding.

### 2.9. Surface plasmon resonance analysis of gp140 antigenic site exposure

Surface plasmon resonance (SPR) analysis was performed on a BIAcore 3000 biosensor (BIAcore, Uppsala, Sweden) at 37°C in HBS-EP buffer (10 mM HEPES, 150 mM NaCl, 0.0005% Tween 20, pH 7.4). 700 resonance units (RUs) of gp140 were coupled to the surface of a CM5 sensor chip using the amine coupling kit according to manufacturer’s instructions (GE Healthcare UK Ltd, Little Chalfont, United Kingdom). Kinetic analysis was performed by injecting serial dilutions of monoclonal antibodies (mAbs) or soluble monomeric CD4 over the chip surface at 50 µl/min for 5 min followed by a 5 min dissociation phase. For mAbs with very slow off-rates, an extended dissociation phase of 30 min was used for the highest two concentrations. The sensor chip surfaces were regenerated between each cycle by injecting 25 µl 10 mM glycine (pH 2). Double referencing was performed using a blank control flow cell as well as a buffer injection, and kinetic rates (k_d_ and k_a_ for dissociation and association rates respectively) were determined by fitting the data to a 1∶1 Langmuir binding model. Dissociation constants (K_D_s) were calculated as KD = k_d_/k_a_.

### 2.10. Estimation of gp140 mix complexity by mass spectrometry

The gp140 immunogens were characterized using tandem mass spectrometry analysis to identify individual gp140 Env peptides within the UG37 and heterotrimeric gp140s. Proteins were deglycosylated using PNGaseF (NEB) using the manufacturer’s instructions, gel electrophoresed and stained with Coomassie blue. Gel bands corresponding to deglycosylated gp140 (75 kDa) were excised and an in-gel trypsin digest was performed as described previously [47]. Sample analysis was performed by nano ultra performance liquid chromatography tandem mass spectrometry (UPLC-MS/MS) on a Waters Q-Tof Premier mass spectrometer (Waters) coupled to a nano-UPLC system (NanoAcquity, Waters) using a reversed phase 75 µm x 250 mm C18, 1.7 µm particle size reversed phase column as described [48]. Peptides and proteins were identified by Mascot (v2.3.01 CBRG Cluster) via automated database searching of all MS/MS spectra against a custom made database including all gp140 protein sequences derived from the constructs used in this study. The data were analyzed with the following search parameters: Type of search: MS/MS Ion Search; Enzyme: Trypsin; Fixed modifications: Carbamidomethyl (C); Variable modifications: Oxidation (M), Deamidated (NQ); Mass values: Monoisotopic; Protein mass: Unrestricted; Peptide mass tolerance: ±0.1 Da (# 13C = 1); Fragment mass tolerance: ±0.2 Da; Max missed cleavages: 2; Instrument type: QTOF. Peptide sequences were compared to a database of the Env proteins used in expression and a score of the −10 log probability of a particular Env sequence being identified was calculated. Taking the set of peptides corresponding to a given Env sequence, the % coverage was derived for each identified Env protein. Due to the similarities between the different gp140 sequences, MS/MS spectra assignments to sequences were evaluated manually for all cases in order to provide the information presented in S2 Table.

### 2.11. Animals

Fifteen Indian rhesus macaques (*Macaca mulatta*) previously vaccinated with HIVconsv antigens aiming to elicit CD8 and CD4 T-cell responses to conserved parts of the HIV proteome [49] were used in our experiment. No significant Env-specific T-cell or B-cell responses were detectable at the time of DNA immunization and no neutralizing antibody titers were detailed at any previous time point. All the animals in the study were housed and cared for following the UK’s code of practice for non-human primates. The animals were housed in a conventional facility in social groups (of 7+) for the duration of the study. The light cycle is 12 hrs dark and 12 hrs light and the switch from light to dark and vice versa occurred gradually. The enclosures are 2.2 m high and the allocated area per animal was between 1.4 to 2.4 m^2^. The enclosures have a variety of semi-permanent structures such as perches, swings and ladders at different heights and windows to the corridor as well as plenty visual barriers.

Other forms of enrichment that are provided routinely include deep bedding (wood shavings) layered to facilitate foraging, toys (kongs, balls, *etc*), destructible items (paper bags, cardboard and wood), foraging devices (puzzles, boxes), frozen treats, baths and daily interaction with the animals care staff. Daily feeding includes at least fruit/veg, primate chow (mash form with seeds and raisins) and two portions forage mix (scattered on bedding). Water was provided ad lib. Animals were trained by positive reinforcement to approach the front of the cage for close inspection and to present and allow topical application of cream on cue. This facilitated thorough monitoring of the vaccination sites and well as minimized the need for restraint.

Animals were purposely bred and used in the UK. A health assessment was carried by the welfare officer and veterinarian at the supplying establishment prior to shipment and also at the scientific establishment on arrival. Daily checks by experienced staff were carried out at least twice per day. Monitoring and scoring systems were used to assess the reaction at vaccination sites. Any reactions where itchiness/discomfort was suspected were reported straightaway to the welfare officer add veterinary surgeon and such animals were treated with NSAIDs. There were no direct adverse effects of the procedure other that the local reactions at the site of vaccination. All animals acclimatised well to the procedures and were keen to approach staff and take treats after recovery from blood sampling/vaccinations. Chemical restraint via use of tranquilizers was utilised to avoid stress/injury (as manual handling expertise was not available at the time), as detailed below. The general behaviour and health of the animals remained mostly stable throughout the study and all the animals were released back to stock at the end of the study and under the advice of the welfare officer and the veterinary surgeon.

### 2.12. Immunizations

All animals were male and between two to three years of age at the start of the immunization schedule and weighed between 5–7 kg. The animals were assigned into three groups of five animals each. All animals were immunised under sedation or anaesthesia during the day and in the dedicated animal laboratory within the housing facility. Intradermal DNA/electroporation was performed according to the Derma Vax protocol (formerly Cyto Pulse Sciences, now Cellectis Therapeutics, Paris, France) and subcutaneous protein and ribi/carbopol boosts were administered in similar locations to the DNA/electroporation prime to act on the nearby draining lymph nodes. Group 1 received the heterotrimeric gp140 with RIBI (RIBI Adjuvant System; Funakoshi Co. Ltd. Tokyo, Japan) adjuvant, Group 2 received UG37 gp140 adjuvanted with RIBI adjuvant and Group 3 received UG37 gp140 with carbopol 974P adjuvant. Animals were anaesthetized only for the purpose of immobilization during vaccination or phlebotomy using ketamine (10 mg/kg) with optional Isoflurane (5 mg/kg). They were vaccinated with the following regimens DDPPP where D is 600 µg of plasmid DNA in 600 µl of saline, and P is 600 µg gp140 protein in 600 µl saline adjuvanted with either an equal volume of RIBI or 1% (v/v) carbopol. The DNA vaccine was delivered i.d. at six sites (each arm, each thigh, one upper and one lower back; 100 µg at each site) with electroporation immediately after injection. A needle array electrode containing two parallel rows of four 2 mm pins (1.5×4 mm gaps) was used and was performed using the Derma Vax DNA Vaccine Skin Delivery System. Protein immunizations were also performed at these six sites (100 µg gp140 protein per site). For the heterotrimer gp140 mixes, the different mixes were rotated between the six injection sites to enhance exposure of the local draining lymph nodes to a wider range of Env gp140 heterotrimer diversity. Macaque blood was drawn from superficial leg veins at the time of each immunization and two weeks after each protein boost. Blood samples were kept at room temperature and were processed within 2 hours of sampling. Serum was separated by centrifugation at 2500 rpm for 10 min in vacutainers (BD Biosciences, San Jose, CA) and stored at −80°C until required. All animal procedures and care strictly conformed to UK Home Office Guidelines. Experiments were conducted according to the National Center for the Replacement, Refinement, and Reduction of Animals in Research, according to EU Directive 2010/63/EU.

### 2.13. Analysis of gp140-specific antibodies in macaque sera

End-point titers were determined as previously described [50]. Briefly, antigens (1 µg/ml in 0.1 M NaHCO_3_, pH 8.5) were used to coat wells of ELISA plates (Greiner Bio-One) at 4°C overnight. Wells were blocked with 2% milk in PBST and incubated with serial dilutions of serum in sample buffer (1% BSA in PBST) for 1 h at RT followed by labelling with goat anti-human IgG-HRP (1∶10,000 in sample buffer; Jackson ImmunoResearch) and developing with 1-Step UltraTMB-ELISA Substrate (Thermo Scientific). The reaction was stopped by addition of 0.5 M H_2_SO_4_ and absorption was read at 450 nm. Data were fitted to a sigmoidal dose-response curve in GraphPad Prism v5.0 and end-point titers were defined as the serum dilution at which the fitted curve reached a value of 0.01 which was more than two standard deviations above the background of no serum control wells.

### 2.14. Viral neutralization assays

Neutralizing antibody activity was measured in 96-well culture plates by using Tat-regulated luciferase (Luc) reporter gene expression to quantify reductions in virus infection in either TZM-bl or A3R5 cells. Assays in TZM-bl cells were performed with Env-pseudotyped viruses as described previously [51]. TZM-bl is a genetically engineered HeLa cell line (also known as JC53-BL) that expresses the CD4 receptor and the CCR5 and CXCR4 coreceptors [52] and contains Tat-regulated reporter genes for firefly Luc and *Escherichia coli* β-galactosidase under regulatory control of an HIV-1 long terminal repeat sequence [53]. The cells were obtained from the NIH AIDS Research and Reference Reagent Program, as contributed by John Kappes and Xiaoyun Wu. Neutralization assays were performed with heat-inactivated (56°C, 1 hr) serum samples, tested at 3-fold dilutions ranging from 1∶20 to 1∶43,740. Diluted samples were pre-incubated with virus (∼150,000 relative light unit equivalents) for 1 hr at 37°C before addition of cells. Following 48 hr incubation, cells were lysed and Luc activity determined using a microtiter plate luminometer and BriteLite Plus Reagent (Perkin Elmer). Neutralization titer (IT50) is the sample dilution at which relative luminescence units (RLU) are reduced by 50%, compared to RLU in virus control wells after subtraction of background RLU in cell control wells. A3R5 (A3.01/R5.6) is a derivative of the CEM human lymphoblastoid cell line that naturally expresses CD4 and CXCR4 [54] and was engineered to express CCR5 [55]. The A3R5 assay was performed with Env.IMC.LucR viruses [56] because multiple rounds of replication are needed to achieve an adequate infection signal in this assay [55]. As with the TZM-bl assay, diluted samples were incubated with virus (∼50,000 RLU equivalents) for 1 hr at 37°C prior to adding cells. After incubating for four days, a defined portion of the cell suspension was transferred to 96-well white solid plates (Costar, Washington, DC) for measurement of luminescence using the ViviRen Live Cell Substrate as described by the supplier (Promega, Madison, WI). For each animal, pre- and post-immune serum samples were assayed side-by-side. Post-immune samples were scored positive for neutralizing antibody activity if the neutralization titer was ⩾3x that of the pre-immune sample.

### 2.15. Competition ELISAs

For cross-competition ELISA, UG37 gp140 was coated onto 96-well plates, washed and blocked as in a standard ELISA. Titration series of macaque sera were added to the wells, directly followed by biotinylated mAbs at their respective 50% binding concentration and the plates were briefly shaken. After 2 hr incubation at RT, plates were washed three times and streptavidin-biotinylated HRP (GE Healthcare) at 1∶1000 in blocking buffer was used for detection as described previously. Data were background-subtracted and normalized to control wells not containing competing sera. 50% inhibition titers (IT50) were determined by fitting a three-parameter dose response curve to the data in GraphPad Prism v5.0 under restriction of the ‘Top’ value to 1.0. IT50 values were defined as the serum dilution at which the values of the fitted curve were reduced by 50%.

### 2.16. Statistical analysis

All statistical analyses were conducted using Graph Pad Prism v5.0. Where data conformed to a Gaussian distribution (as tested by the D′Agostino & Pearson omnibus normality test) and variances did not differ (as tested by Bartlett’s test for equal variances), parametric ANOVAs were selected; where data did not fit a Gaussian distribution, non-parametric tests were selected. The gp140 ELISA data were analyzed using ANOVAs with Bonferroni post hoc tests on log-transformed data. Comparisons between different time-points for each group in the UG37 ELISA were made using one-way ANOVAs; comparisons between groups in the protein mix ELISAs were made using two-way ANOVAs. For the neutralization data, a Kruskal-Wallis test was performed for each pseudovirus with Dunn’s post-hoc tests to compare each of Group 1 and Group 3 with the control Group 2. Comparisons of composite neutralization data between Groups 1 and 3 with Group 2 were made using non-parametric Mann Whitney tests. For the competition ELISAs a one-way ANOVA was performed for each Ab on log-transformed data with Dunnett’s post-hoc tests to compare Group 1 and Group 3 with the control Group 2. All tests were two-sided and the post-hoc analyses incorporated corrections for multiple tests. Significance was determined at *p<0.05, **p<0.01, ***p<0.001, and ****p<0.0001.

## Results

### 3.1. Isolation, screening and selection of patient-derived env genes

Sera from 84 HIV-1-positive individuals collected by the Centre for AIDS Reagents at the NIBSC were included in an initial screen of *env* sequences. These included individuals from diverse geographic regions who were infected with HIV-1 of subtypes A, B, C, D, F and CRF_AE, and who displayed varying levels of bNAbs in their sera (data not published). Viral RNA was extracted from patient sera and *env* genes amplified and sequenced. A total of 661 sequences were obtained from 58 individuals with an average of 11 sequences per patient (range 2–35). From these, 224 *env* genes were cloned into the pLEXm protein expression vector and screened for their ability to express gp140 following transient transfection of 293 T cells. A total of 54 *env* clones were selected for inclusion in the immunization study based on their protein expression ability and on their phylogenetic distribution (Fig. 1). These formed five groups of envs with the following compositions: Mix 1 comprised 15 subtype A; Mix 2 comprised 9 subtype B; Mix 3 comprised 10 subtype B (herein referred to as subtype B′ to differentiate from Mix 2); Mix 4 comprised 11 subtype C; Mix 5 comprised 9 subtype D and F.

### 3.2. Production of heterotrimers following co-transfection of multiple Env expression constructs

**Figure 2:**
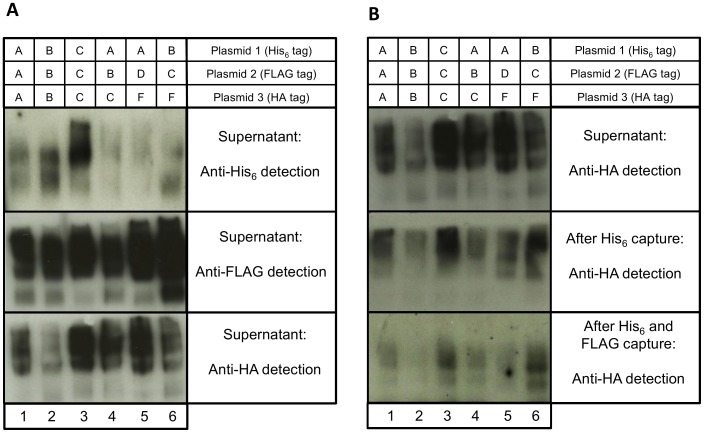
Non-reducing SDS-PAGE and western blot analysis of gp140 expression following co-transfection of multiple *env* plasmids. Six flasks were transiently transfected with three different gp140 *env* plasmids. 1∶3x subtype A, 2∶3x subtype B, 3∶3xsubtype C, 4: subtypes A, B and C, 5: subtypes A, D and F, 6: subtypes B, C and F. (**A**) Detection of gp140 proteins in supernatant with anti-His_6_, anti-FLAG and anti-HA antibodies. All three tags were detected for each of the six *env* combinations. (**B**) Detection of HA-tagged gp140 in transfection supernatant, after His_6_-capture with Co^2+^ beads, and after FLAG-capture with anti-FLAG-tagged magnetic beads. The HA tag was detectable at all stages in all six transfections.

A proof-of-concept experiment was set up to identify heterotrimer formation both within and between HIV-1 subtypes. Six cultures of 293 T cells were each transiently transfected with three env expression constructs containing different N-terminal tags: one His_6_, one FLAG and one HA, and each encoding a different Env. Flasks 1–3 were transfected with three env genes of the same HIV-1 subtype: A, B, or C, respectively. Flasks 4–6 were transfected with three env genes of different subtypes (Flask 4: subtypes A, B and C; Flask 5: subtypes A, D and F; Flask 6: subtypes B, C and F). After 48 hr, supernatants were collected and screened for expression of gp140 by western blot using antibodies against His_6_, FLAG and HA (Fig. 2A). In all six combinations, the three tags were detected, demonstrating the co-expression of multiple gp140s in the transfection supernatants, although the ratios of the tags did vary across samples.

To prove true heterotrimers had formed and all three tags were present within individual trimers, a two-stage capture experiment was conducted. Supernatant was first passed over a Talon (Clontech) cobalt chloride metal-affinity column to specifically bind His_6_-tagged protein, capturing gp140s with at least one His_6_-tagged monomer. The eluates from this column were then incubated with magnetic beads coated with anti-FLAG antibodies, thus capturing trimers that had both the His_6_ and FLAG tags. Eluted proteins were analyzed by western blotting with an HA antibody to detect the presence of the third tag (Fig. 2B). The HA tag was detected in all six supernatants following both His_6_ and FLAG capture, demonstrating the ability of heterotrimers to form, both within and between HIV-1 subtypes. However, levels were low, indicating that these heterotrimers may be quite rare. Controls were included to ensure the specificity of antibody detection by western blot and the specificity of binding to both the Talon cobalt chloride resin and the anti-FLAG-tagged magnetic beads (S1 Figure).

### 3.3. Purification and antigenic characterisation of gp140 protein immunogens

**Figure 3:**
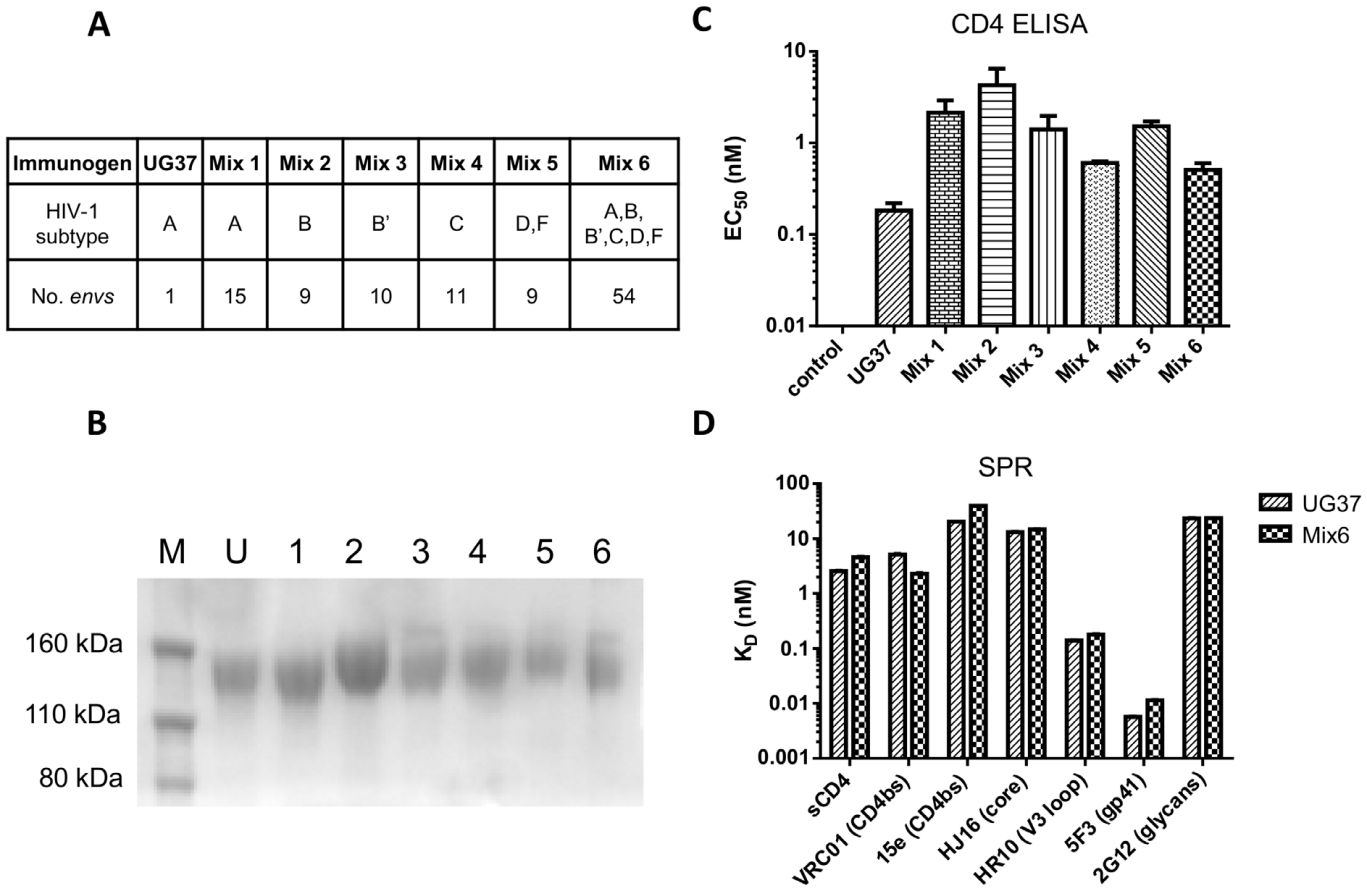
SDS-PAGE and antigenic verification of gp140 protein immunogens. (**A**) Components of immunogens; protein immunogens were derived by co-transfection of multiple gp140 *env* plasmids. Mix 1: subtype A; Mix 2: subtype B; Mix 3: subtype B′; Mix 4: subtype C; Mix 5: subtype D and F; Mix 6: all *envs* from Mixes 1 to 5. (**B**) SDS-PAGE analysis of gp140 immunogens. Proteins were separated under reducing conditions and stained with Coomassie Blue. M: Protein marker, U: UG37, 1–6: Mixes 1–6. Bands correspond to gp140 monomers. (**C**) ELISA analysis of tetrameric CD4-IgG2 binding to all gp140 immunogens. Values shown are the concentrations of CD4-IgG2 required to reach 50% of the maximum signal. (**D**) Surface plasmon resonance (SPR) analysis of a panel of monoclonal antibodies and monomeric soluble CD4 binding to UG37 and heterotrimer Mix 6. Epitopes targeted are specified in parentheses. Dissociation constants are derived from a kinetic analysis of the binding curves.

The principal hypothesis of this study is that the large antigenic diversity achieved through the immunization with polyvalent heterotrimeric gp140 mixtures may result in NAb responses of greater breadth and potency as compared to a single homotrimeric gp140. This would be either from focusing on epitopes conserved between Envs or an expansion of multiple B-cell specificities to the spectrum of epitopes presented by immunization. Having shown the feasibility of production of heterotrimers by co-transfection of multiple gp140 *env* expression plasmids, a series of *env* plasmid combinations was designed for a macaque immunization study. A total of 54 *env* plasmids with good expression levels and of diverse genetic representation were selected by phylogenetic analysis for expression of six immunogen mixtures (Fig. 3A; Mixes 1–6).

Following large-scale transfection the gp140 trimers were purified to >95% purity for immunization studies. First, the His_6_-tagged gp140 was captured by a Talon cobalt chloride metal-affinity column and the eluted proteins were further purified by SEC to resolve the different oligomeric forms: aggregates, trimers, dimers and monomers. Fractions corresponding to the trimer peak were further purified using GNA-lectin affinity capture, which binds specifically gp140, and this was followed by an additional SEC step which resulted in a well resolved single peak with a molecular weight corresponding to the trimer. The purity of the gp140 immunogens was assessed by SDS-PAGE under reducing conditions; all proteins separated into monomeric gp140s in the presence of DTT (Fig. 3B).

The accessibility of the CD4bs on the gp140 trimers was verified by ELISA using a soluble tetrameric human IgG2-CD4 fusion protein. All seven trimer preparations bound with high affinity to soluble IgG2-CD4 with ELISA-derived EC_50_s ranging from 0.18 nM for UG37 to 4.28 nM for Mix 2 (Fig. 3C). Binding properties of UG37 and Mix 6, the latter containing all 54 Env proteins, were further analyzed by SPR (Fig. 3D). This method confirmed binding of soluble monomeric CD4 (sCD4) to both antigens and in addition showed binding to a panel of mAbs targeting the CD4bs (VRC01 and 15e), the core epitope (HJ16), the V3 loop (HR10), gp41 (5F3), and the glycan shield (2G12). The mAbs tested bound with a broad spectrum of SPR-derived K_D_s ranging from 0.01 nM to 40 nM for mAbs 5F3 and 15e, respectively.

To verify the diversity of the gp140 mixtures expressed from cells, we addressed expression of individual Env proteins using tandem mass spectrometry (MS). S2 Table shows all gp140 sequences identified by MS as well as the assignment of unique peptide sequences that relate to each gp140 genotype. The compilation of peptide ‘signature’ sequences unique to each expected Env genotype in the co-transfected DNA plasmid mixtures confirmed that between 30% (Mix 3) and 71% (Mix 1) of Env genotypes could be positively identified. This clearly indicated that a large proportion of the gp140 genotypes were co-expressed and purified in the final protein immunogens, thus confirming the production of novel gp140 immunogens containing substantial protein diversity. It is possible that for peptides that were not detected, the fragments could have had reduced ionization efficiency or confounding masses, or were produced at lower levels such that they escaped MS based detection.

### 3.4. Immunization of macaques with gp140 proteins

**Figure 4:**
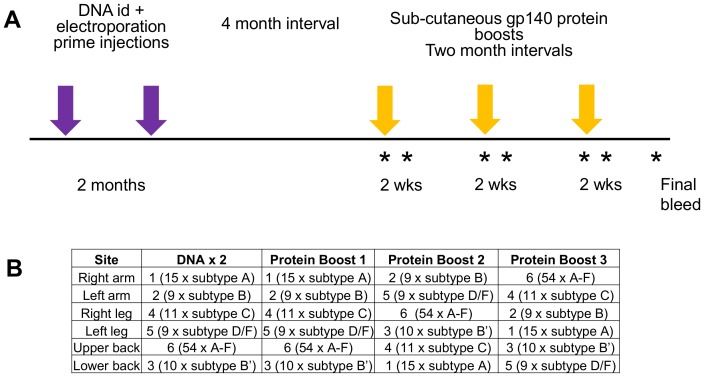
Immunization schedule. (**A**) Timeline of DNA and protein immunizations. Macaques were each given two DNA priming injections spaced by two months followed by a four-month interval and then three gp140 protein boosts, spaced by two-month intervals. Blood was collected at the time of each protein boost and two weeks after each boost (*). A final bleed was collected eight weeks after the final boost. (**B**) Each immunization consisted of six separate injections at different sites on the macaques. In the heterotrimer group the six different immunogens were rotated around these sites so that any local draining lymph nodes experienced different immunogens after each injection. Details given here indicate which Mix immunogen was administered at each site (1–6) and the contents of those mixes; ‘15 × subtype A’ indicates that Mix 1 contains heterotrimers generated from 15 subtype A Envs.

Fifteen male rhesus macaques were immunized with the gp140 trimers (Fig. 4), in three groups of five macaques to compare (i) heterotrimeric gp140 mixtures with homotrimeric UG37 gp140 (Group 1 *vs*. Group 2), and (ii) RIBI with carbopol 974P (Group 2 *vs*. Group 3). Group 1 received the polyvalent gp140 heterotrimer mixtures adjuvanted with RIBI (Heterotrimer RIBI). Group 2 received homotrimeric UG37 gp140 adjuvanted with RIBI (UG37 RIBI). Group 3 macaques received UG37 gp140 adjuvanted with carbopol (UG37 carbopol).

The immunization schedule consisted of two DNA gp140 primes which had been shown to assist in the development of more robust neutralizing responses when followed by a protein boost [57], spaced by two months and followed by a four month resting period and then three protein boosts at two or three month intervals (Fig. 4A). Blood samples were collected at each immunization and two weeks after each protein boost. Each boost consisted of six separate injections at different anatomical sites. For Group 1, each of the six injections consisted of one of the six different gp140 mixes (Fig. 4B); these were rotated between the six sites so that local draining lymph nodes would encounter gp140 proteins of different HIV-1 subtypes throughout the study. Overall, carbopol was better tolerated than RIBI in the macaques, with the latter adjuvant producing some local swelling and occasional ulceration.

### 3.5. All macaques generated gp140-specific binding antibodies following gp140 trimer immunization

**Figure 5:**
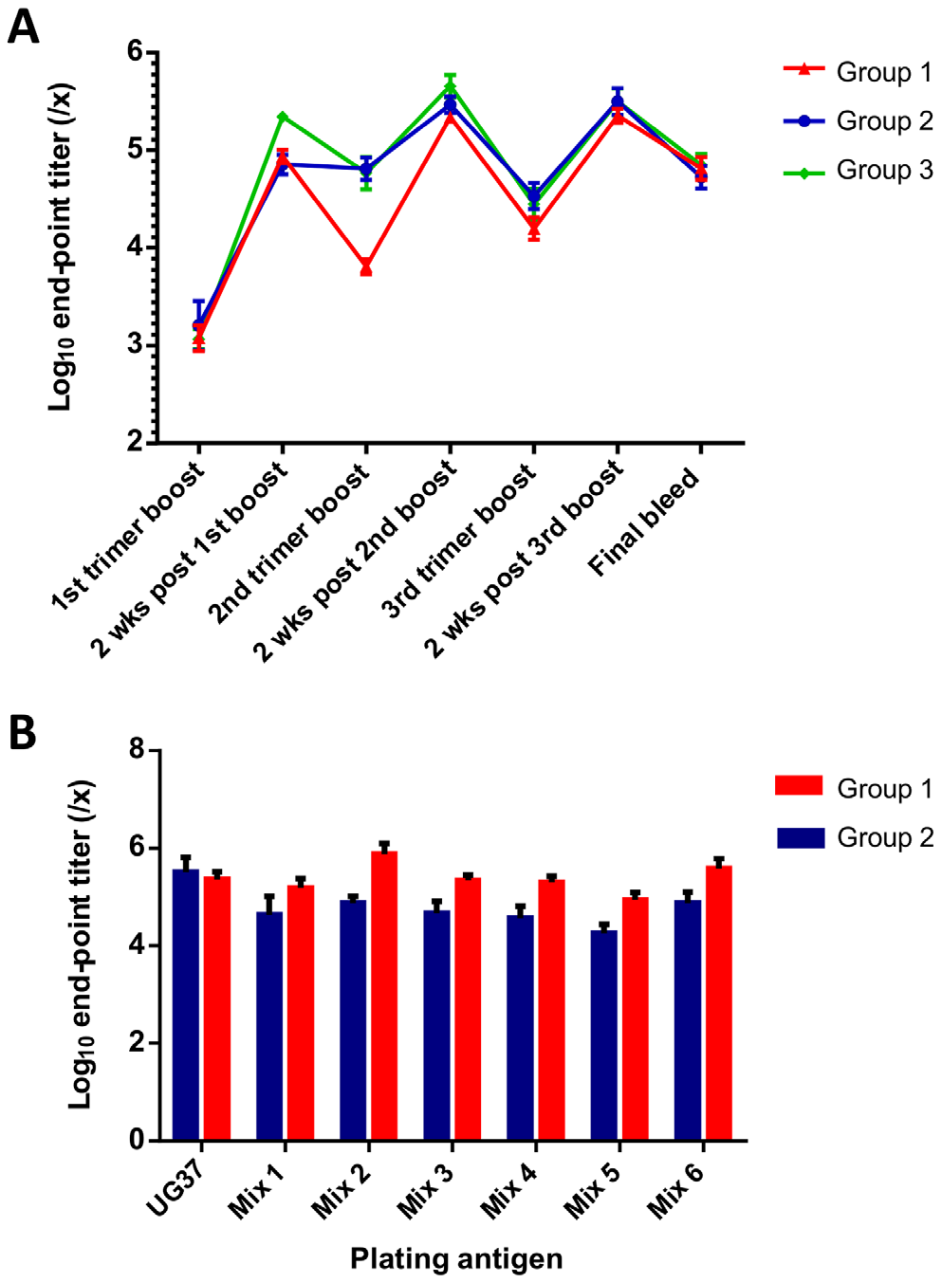
ELISA analysis of gp140-specific antibody levels in macaque sera. (**A**) Log_10_ end-point ELISA titers assayed against UG37 gp140 at the time of each trimer protein boost, two weeks after each protein boost, and at the final bleed. Titers are represented as serum dilution factors (1/x). Group mean titers are plotted with error bars to indicate the standard error of the mean. (**B**) Mean log_10_ end-point titers against UG37 and individual protein mixes (Mixes 1–6) for Group 1 (Heterotrimer, Ribi) and Group 2 (UG37, Ribi) macaques two weeks after the third protein boost. Error bars show standard error of the mean. For both, end-point titers were defined as the serum dilution at which the fitted sigmoidal dose-response curve reached an OD_450_ of 0.01.

Levels of gp140-specific binding antibodies in sera from all macaques were analyzed by end-point ELISA using plates coated with UG37 gp140 (Fig. 5A). Macaques in all groups generated robust gp140-specific antibody responses detectable two weeks after the first protein trimer immunization which were boosted further following the second protein immunization, however the third boost did not show a significant increase in gp140-binding titers. In all three groups, antibody binding titers two weeks after each protein immunization were significantly greater than titers at the time of immunization (p<0.0001). Data for individual macaques at each time-point are given in S3 Table.

Between protein immunizations, a significant decline (up to 10-fold from peak) in titers occurred in all macaques (Fig. 5A). Persisting titers between administrations were higher at the earlier time-points in Groups 2 and 3 than in the Group 1 macaques, but the titers post final boost were equivalent in all groups (Fig. 5A).

Peak gp140 Ab titers two weeks after each trimer administration were slightly higher in Groups 2 and 3 than in the Group 1 macaques (Fig. 5A), though the differences did not reach statistical significance at any time-point (*p*>0.05). These differences could reflect the use of UG37 gp140 as the plating antigen for the ELISA, as Group 1 macaques did not receive this trimer in their immunizations, and ELISA titers for this group comprise UG37 gp140 cross-binding titers.

To determine whether gp140-binding Ab titers were actually lower in Group 1 macaques than in those macaques that had received UG37 immunogens (Groups 2 and 3) or whether this was due to the plating antigen being UG37, serum samples from Groups 1 and 2 were also tested for binding against the individual protein mixtures (Fig. 5B). Two weeks after the third trimer boost, peak titers were significantly higher in Group 1 macaques than in Group 2 macaques for all mixes (Mix 1: *p*<0.05; Mix 2: *p*<0.001; Mix 3: *p*<0.0001; Mix 4: *p*<0.01; Mix 5: *p*<0.0001; Mix 6 *p*<0.001). Interestingly, the difference was least marked for Mix 1, perhaps due to the immunogen UG37 being of the same HIV-1 subtype as the plating antigen in that case. For both groups of macaques, the binding titers were variable, but present against all mixes.

### 3.6. Breadth and potency of neutralization was slightly improved in macaques that received heterotrimers or UG37 with carbopol compared to UG37 RIBI

To examine the breadth of neutralization a standardized panel of tier 1 pseudoviruses in a TZM-bl assay was used to assess sera taken two weeks after the third protein boost. Neutralization of six tier 1 pseudoviruses of subtypes B, C and CRF01_AE was observed; results are shown as reciprocal serum dilutions able to achieve 50% neutralization of a given pseudovirus (Table 1). Group 1 showed neutralizing titers against six pseudoviruses whereas groups 2 and 3 showed neutralization of five pseudoviruses.

**Table 1:** ID_50_ tier-1 pseudotyped virus neutralization titers in the TZM-bl assay.

		Env-pseudotyped viruses					
Immunogen	Macaque	SF162	BX08	92BR025.9	MN.3	MW965.26	TH023.6
**Heterotrimer**	1	**845** #	**25**	21	**1453**	949	428
**RIBI**	2	**150**	20	29	**680**	2004	1201
**(Group 1)**	3	**3880**	**37**	29	**5417**	**6016**	269
	4	**4026**	**50**	20	**7471**	**5792**	**3275**
	5	**223**	**25**	**64**	**844**	**9888**	**2558**
	Group mean	1825	31	33	3173	4930	1546
	6	24	20	**57**	191	1795	274
**UG37**	7	31	20	20	120	**3140**	600
**RIBI**	8	50	20	**41**	464	1259	**1137**
**(Group 2)**	9	56	20	36	225	2498	**1534**
	10	76	20	31	318	2358	337
	Group mean	48	20	37	264	2210	776
	11	20	20	25	25	1997	**2991**
**UG37**	12	**891**	20	**89**	**477**	**27264**	**9869**
**Carbopol**	13	68	20	**66**	361	2164	**1862**
**(Group 3)**	14	**153**	20	**61**	**691**	**5543**	**1728**
	15	124	20	**42**	160	**3759**	759
	Group mean	251	20	57	343	8146	3442
***Median*** $		***124***	***20***	***36***	***464***	***2498***	***1201***

# Numbers given are the sample dilutions at which relative luminescence units (RLU) were reduced by 50% compared to RLU in virus control wells after subtraction of background RLU in cell control wells. All samples were taken two weeks after the third protein boost.

$ Values above the median titer for each pseudovirus are shown in bold.

For each pseudovirus, the median neutralization titer was calculated. Thus, neutralization of MN.3 (subtype B), MW965.26 (subtype C), and TH023.6 (CRF01_AE) was achieved in all macaques of all groups, an unsurprising result given their relative sensitivity to neutralization. Both the Group 1 (20 data points greater than median) and Group 3 (15 data points greater than median) showed better overall neutralization than the Group 2 macaques (5 data points greater than median). Additionally, titers were significantly greater, indicating a greater potency in Group 1 compared to Group 2 for SF162 (*p*<0.01), BX08 (*p*<0.05) and MN.3 (*p*<0.05). None of the differences between Group 2 and 3 macaques reached statistical significance. A composite analysis of the neutralization data from all pseudoviruses confirmed statistical significance for the marginally greater titers of Group 1 (*p* = 0.0473), but not Group 3 as compared to Group 2 control macaques (S2 Figure).

Neutralization of tier 2 pseudoviruses on the same serum panel was detected in the more sensitive A3R5, but not in the TZM-bl neutralization assay (Table 2). Again, all macaques were able to neutralize some IMC.LucR viruses from the panel, although for many, the titers were only slightly above the baseline of the assay. Both Groups 1 and 3 showed greater breadth of neutralization than the Group 2 macaques. In this assay, titers were significantly greater in Group 1 as compared to the Group 2 controls for CAP45 (subtype C; *p*<0.05) and RHPA (subtype B; *p*<0.05), whilst titers were significantly greater in Group 3 as compared to the Group 2 controls for CAP45 (subtype C; *p*<0.01), Ce1176 (subtype C; *p*<0.05) and Du151 (subtype C; *p*<0.05). Analysis of composite neutralization titers showed greater overall potency in Group 3 macaques as compared to Group 2 (*p* = 0.0138), with Group 1 not reaching statistical significance. Together, these results indicate that the Group 1 and 3 immunogens generate slightly greater breadth and potency of neutralization than the Group 2 control immunogen.

**Table 2:** ID_50_ tier-2 pseudotyped virus neutralization titers in the A3R5 assay.

		Env-pseudotyped viruses							
Immunogen	Macaque	CAP45	Ce1086	Du151.2	Ce1176_A3	C1080.c03	R2184.c04	SC22.3C2	RHPA
	1	**73** #	64	48	**44**	161	36	218	**317**
**Heterotrimer**	2	66	51	52	37	**596**	**53**	**520**	**354**
**RIBI**	3	**95**	**104**	56	**43**	418	**64**	312	**373**
**(Group 1)**	4	62	35	43	25	522	40	334	**306**
	5	**145**	**164**	**75**	**46**	**854**	**90**	**1624**	**347**
	Group mean	88	84	55	39	511	57	602	339
	6	20	**95**	54	39	206	35	**470**	145
**UG37**	7	20	52	47	22	204	26	149	61
**RIBI**	8	20	94	61	33	554	**58**	356	217
**(Group 2)**	9	20	72	40	25	**991**	43	386	147
	10	20	75	**71**	37	332	**57**	**663**	275
	Group mean	20	78	55	31	457	44	405	169
	11	**72**	69	**62**	**52**	**1781**	44	287	113
**UG37**	12	**147**	**203**	**78**	**48**	**2822**	41	**507**	**459**
**Carbopol**	13	63	**219**	**76**	39	**929**	35	369	215
**(Group 3)**	14	**130**	**245**	**77**	**55**	**1329**	**86**	**509**	**358**
	15	**72**	**387**	**63**	38	433	**47**	**427**	146
	Group mean	97	225	71	46	1459	51	420	258
***Median*** $		***66***	***94***	***61***	***39***	***554***	***44***	***386***	***275***

# Numbers given are the sample dilutions at which relative luminescence units (RLU) were reduced by 50% compared to RLU in virus control wells after subtraction of background RLU in cell control wells. All samples were taken two weeks after the third protein boost.

$ Values above the median titer for each pseudovirus are shown in bold.

All neutralization assays were performed on sera from two weeks after the third protein boost where the gp140-ELISA titers were high (Fig. 5A). For four macaques (macaques 2, 5, 9 and 14; Tables 1 and 2), neutralization titers were also determined against the Tier-1 pseudotyped viruses MN.3 and MW965.26 for all time-points using the TZM-bl assay. As expected, titers fluctuated over the course of the study, with peaks in neutralization titers correlating with the peaks and troughs in ELISA titers during the protein boosts (S3 Figure).

### 3.7. Antibodies target multiple regions of Env

**Figure 6:**
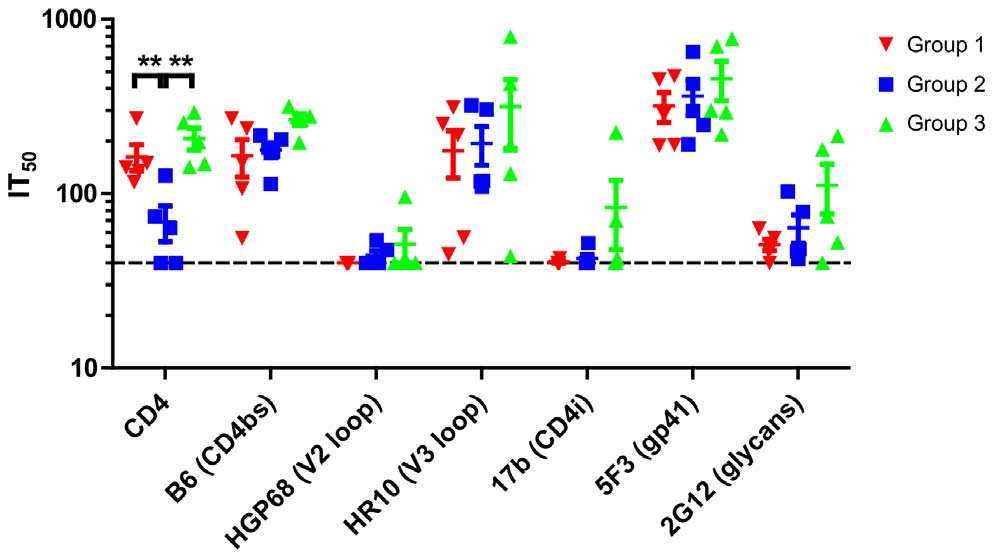
Cross-competition ELISA. Titration series of sera from two weeks after the third protein boost were competed with biotinylated mAbs or biotinylated soluble domains 1–4 monomeric CD4 using UG37 gp140 as the antigen and 50% inhibition titers (IT50) were calculated as the serum dilution at which binding of the biotinylated probes was reduced by 50% compared to control well without serum. Epitopes targeted by the mAbs are specified in parentheses. CD4bs: CD4 binding site; CD4i: CD4 inducible epitope. A one-way ANOVA was performed for each competing protein/antibody with Dunnett’s post-hoc tests to compare Group 1 and Group 3 with the control Group 2. Significance is illustrated by **p<0.001.

Cross-competition ELISA was used where antibodies in the sera competed for gp140 binding with sCD4 and a panel of mAbs to map the binding specificities of the serum antibodies. This assay was performed on both pre-immune serum and serum taken two weeks after the third (final) protein boost. None of the pre-immune sera competed with any of the probes in this assay (data not shown). In contrast, sera collected two weeks after the final immunization showed considerable competition with sCD4 and the CD4bs antibody B6. The former was significantly higher in both the Group 1 and 3 macaques compared to the Group 2 control macaques (*p*<0.01, Fig. 6). In all macaques, competition was detected against the V3-loop antibody HR10 and the gp41-targeting mAb 5F3, but not against the V2-loop-directed mAb HGP68 (Fig. 6). Five macaques showed weak competition against the CD4-induced (CD4i) epitopes of mAb 17b, indicating some exposure of CD4i epitopes, which may have arisen from the binding of gp140s to endogenous macaque CD4 on immunization. Additionally, competition was detected with 2G12, suggesting either the presence of glycan-specific antibodies in the macaque sera or antibodies overlapping the 2G12 binding site (Fig. 6).

Overall, the level of competition varied between macaques, although all macaques generated antibodies that targeted multiple epitopes on gp140. Additionally, targeting of the CD4bs was slightly improved in both HR and UC macaques, compared to the UG37 RIBI control group.

## Discussion

One of the major challenges for HIV-1 vaccine design is to tackle the enormous genetic diversity of the virus by creating an immunogen that elicits bNAbs. It may be necessary for a successful vaccine regimen to replicate the chronic exposure experienced in natural infection during which somatic mutation of Env-specific antibodies may lead to their increased neutralizing capacity [17], [58], potentially resulting in NAbs of great potency and breadth [20], [26], [39]. Some polyvalent vaccine regimens involving the co-administration of multiple monomeric gp120 [27], [29], [59] or gp140 [30] Env proteins generated improved NAb responses in comparison to monovalent equivalents, as did the sequential administration of different gp140 *env* DNA constructs [32]. Alternative approaches sought to focus the B-cell response through the presentation of conserved Env regions or consensus or founder sequences [60]–[63], with some success at inducing cross-reactive NAbs. In the current study, we aimed to maximise the representation of antigenic diversity of gp140 antigens through the generation of heterotrimers from a diverse set of patient-derived Envs, and compared their immunogenicity to homotrimeric UG37 gp140 in macaques. Our strategy incorporated three sources of diversification: the use of 54 unique and genetically diverse envelopes; the generation of gp140 heterotrimers to further increase the number of epitopes displayed on the trimers, and the sequential rotation of heterotrimeric Env deliveries of different subtypes around 6 anatomical sites on the macaques.

Sellhorn *et al*. described the generation of heterotrimers for which co-transfection of two *env* expression constructs (subtype A and subtype B: SF162) was performed in 239T cells [33]. The authors demonstrated the immunogenicity of their heterotrimers in rabbits using a DNA-prime and protein-boost regimen, and observed induction of antibodies with improved breadth of neutralization with the heterotrimers than with homotrimeric (A/B-subtype) gp140. Here, we adopted a similar approach to generate heterotrimers, but the proof-of-concept sequential capture experiments extended to demonstrating the presence of three different protomers from three different subtypes (A, B and C; A, D and F; B, C and F) within a given HIV-1 trimer. This builds on the work of Sellhorn and colleagues, and establishes a principle whereby further increasing the diversity of a polyvalent HIV-1 trimeric immunogen is possible. Many of the heterotrimers in the mixes would have contained trimers in which each of the three polypeptides composing the trimer was unique. The predicted result is that the polyvalent heterotrimers could bind B-cells with cross-reactive BCRs more effectively than B-cells specific to individual hypervariable regions. Indeed, individual protomers of the heterotrimers with three distinct gp140s should trivalently bind better to B-cells with cross-reactive BCRs specific to conserved epitopes between the three trimer protomers, whilst BCRs with specificity to a single protomer or variable region of the heterotrimer would have lower avidity to the antigen, resulting in lower activation of these B-cell specificities. Six different mixes from *env* constructs were formulated: four mixes contained *env*s from a single HIV-1 subtype (n = 9–15), Mix 5, which contained both D and F, and the final mix which contained all 54 *envs*. By introducing the possibility of forming heterotrimers, the polyvalency of a protein immunogen vastly increases: 10 different *envs* have the potential to form up to 120 different Env trimer assemblies with a small proportion of homotrimers, more heterotrimers with two different protomers, and the majority being heterotrimers with three distinct Env protomers. Although the extent of diversity present in the gp140 heterotrimer immunogens cannot be precisely determined, the presence of many Envs within each immunogen mix was demonstrated using mass spectrometry.

The macaque immunization study revealed a slightly improved neutralization potency of elicited responses when using gp140 heterotrimer mixtures as compared to a monovalent gp140 homotrimer, and also when carbopol 974P was used as an adjuvant rather than RIBI. Levels of total gp140-specific antibodies in the macaque sera, as determined by ELISA, were equivalent for the three groups and were present at levels comparable to, or higher than, those elicited in other immunization studies using gp120 or gp140 protein immunogens [30], [31], [64]–[66]. When using UG37 as the plating antigen, titers appeared to be lower in the HR immunized macaques than in the other groups, but when the heterotrimeric protein mixes were used for plating, titers in HR macaques were equivalent to those seen in the UG37 gp140 ELISA for UR and UC. The NAb titers were similar to those generated from previous gp140 immunizations for the easy-to-neutralize viruses, such as SF162, MN.3 and MW965.26 [30], [31]. However, in contrast to these previous macaque studies, our immunized macaques showed some neutralization of tier 2 viruses. In other challenge studies, these levels of NAbs were sufficient to provide protection against mucosal challenge [10], [13], [14], in which protection correlated with NAb titers [13], [67].

In addition to generating good potency and breadth of NAb response, it is desirable for a vaccine to induce antibodies that target the conserved regions of the Env from which escape is less likely. For example, a number of bNAbs (*e.g.*, VRC01) target the CD4bs [68]–[70] or glycosylated regions of the Env variable loops (*e.g.*, PG9, PGT122, PGT145) [19], [21], [71], [72], and patients with CD4bs Abs are more likely to have broad and potent NAb responses [17], [73]. We used competition ELISA to determine the specificity of the NAb responses in the three macaque groups. All macaques generated NAbs specific to different epitopes across the Env protein, but a marginally improved targeting of the CD4bs with both the heterotrimeric gp140 RIBI or UG37-carbopol groups in comparison with the homotrimeric UG37-RIBI was detected. Although SPR analyses demonstrated comparable binding of soluble CD4 and a panel of mAbs between monomeric UG37 gp140 and the most heterologous immunogen, Mix 6, these results fit with the expectation that the presentation of different epitopes on the heterotrimers results in multiple specificities of the NAbs. Indeed this is in line with observations by Sellhorn *et al*., who reported alterations in exposure of epitopes at the CD4bs, the co-receptor binding-site, and the MPER on their heterotrimers [33]. In the adjuvant comparison study, the UG37 gp140 antigen was identical in the two groups; however an increase in CD4bs titers was observed in the carbopol group. The higher potency of NAb and increase in CD4bs-directed Abs generated by carbopol could be attributed to an improved antigenic domination and specificity of the adaptive immune response, and potentially due to its capacity to induce a strong T-helper response and as an aqueous-based adjuvant that does not interfere with the Env antigenic structure [34].

Despite the generation of high Env-specific IgG titers in the macaques after the first and subsequent protein boosts, a significant decline in titers was observed between protein administrations (up to 10-fold). A similar pattern was previously observed in macaques immunized with Env trimers [66], where the authors also analyzed the kinetics of the B-cell response to the immunogen. They observed a significant increase in peripheral Env-specific antibody-secreting plasma cells (PCs), which peaked 7 days after each protein immunization, but rapidly declined to base levels. As in this study, Sundling and colleagues observed a rapid recall response upon boosting which probably resulted from circulating memory B-cells that differentiated into short-lived PCs [66]. They detected stable levels of memory B-cells between immunizations, but levels would be expected to wane over a longer period of time, as the Ab response against gp120 and gp140 Env is not maintained long-term following clearance of the immunogen [74]–[76]. This phenomenon is not restricted to the vaccination setting, as chronically-infected HIV-1 individuals on ART with suppressed viral loads also experience a decline in their plasma antibody and memory B-cell levels although slower than in the acute phase [76]. Vaccines and infections of other diseases produce long-lived neutralizing memory B-cells central to protection. It is therefore important to understand the exact mechanisms involved in the generation of long-lived memory responses.

In summary, we have demonstrated that it is possible to form heterotrimers with three different Env protomers, both within and between HIV-1 subtypes, and used this strategy to generate highly-diverse immunogens. Exposure of the macaque immune system to these highly diverse gp140 heterotrimer immunogens resulted in the generation of NAb responses of marginally improved breadth and potency in comparison to monovalent homotrimeric gp140 using the same adjuvant and improved targeting of antibodies to the CD4bs. Future studies will need to optimise the heterotrimeric regimen with new HIV-1 trimer and adjuvant technologies that may enable more potent bNAb responses to be elicited. We also demonstrate that carbopol is a safe and effective adjuvant for use in macaques and capable of promoting an improved anti-HIV-1 NAb response compared to RIBI. Further development of these vaccine strategies and testing in a challenge model would be necessary to determine whether they could contribute towards HIV-1 protection.

## Supporting Information

S1 Figure **Western blot analysis to detect specific capture and detection of Env trimers with either one or two tags in series.** Samples were separated by SDS-PAGE and analysed separately by western blot with anti-His_6_, anti-FLAG and anti-HA antibodies. Plasmids: 1 (His_6_), 2 (FLAG), 3 (HA), 4 (FLAG-His_6_) and 5 (HA-His_6_). (i) Rows 1–5: Supernatants following transient transfection with plasmids 1–5; (ii) Rows 6–10: Elutions from Co^2+^ column (specific for His_6_) following capture of supernatants 1–5; (iii) Rows 11–15: Elutions from anti-FLAG-tagged magnetic beads following capture of supernatants 1–5.
(TIF)

S2 Figure **Composite analysis of neutralization data.** Tat-regulated Luc reporter gene expression was determined to quantify reductions in virus infection in either (**A**) TZM-bl or (**B**) A3R5 cells. Neutralization titers are the sample dilution at which relative luminescence units (RLU) were reduced by 50% compared to RLU in virus control wells after subtraction of background RLU in cell control wells. Comparisons between groups were made individually using Mann Whitney tests. Significance at *p*<0.05 is indicated.
(TIF)

S3 Figure **TZM-bl neutralization ID_50_ titers in four macaques throughout the protein immunization time-course.** Macaque 2 (Group 1), Macaque 5 (Group 1), Macaque 9 (Group 2) and Macaque 13 (Group 3). (**A**) MN.3 (tier 1, subtype B). (**B**) MW965.26 (tier 1, subtype C). In both, individual neutralization titers are shown for each of the four macaques on the left-hand axis, and the mean gp140 ELISA titer of these four macaques is shown on the right-hand axis as serum dilution factors (1/x).
(TIF)

S1 Table **Primer details.** Table of primers used for cDNA library formation from purified viral RNA extracted from human serum and amplification of env genes by RT-PCR and PCR for cloning into the expression vector pLEXm.
(DOC)

S2 Table **Characterisation of gp140s by tandem mass spectrometry.** Table of Env peptides sequences derived from tandem mass spectrometry for either UG37 gp140 Env or heterotrimer Env samples.
(DOC)

S3 Table **ELISA titration end-points.** Table of ELISA end point titers for individual macaque sera at all experimental sampling timepoints.
(DOCX)
