# Supplementary figures and images for: Comparison of Neutralizing Antibody Responses Elicited from Highly Diverse Polyvalent Heterotrimeric HIV-1 gp140 Cocktail Immunogens versus a Monovalent Counterpart in Rhesus Macaques
Source: PLoS One. 2014 Dec 9;9(12):e114709. doi: 10.1371/journal.pone.0114709 (PMC4260879; doi:10.1371/journal.pone.0114709)

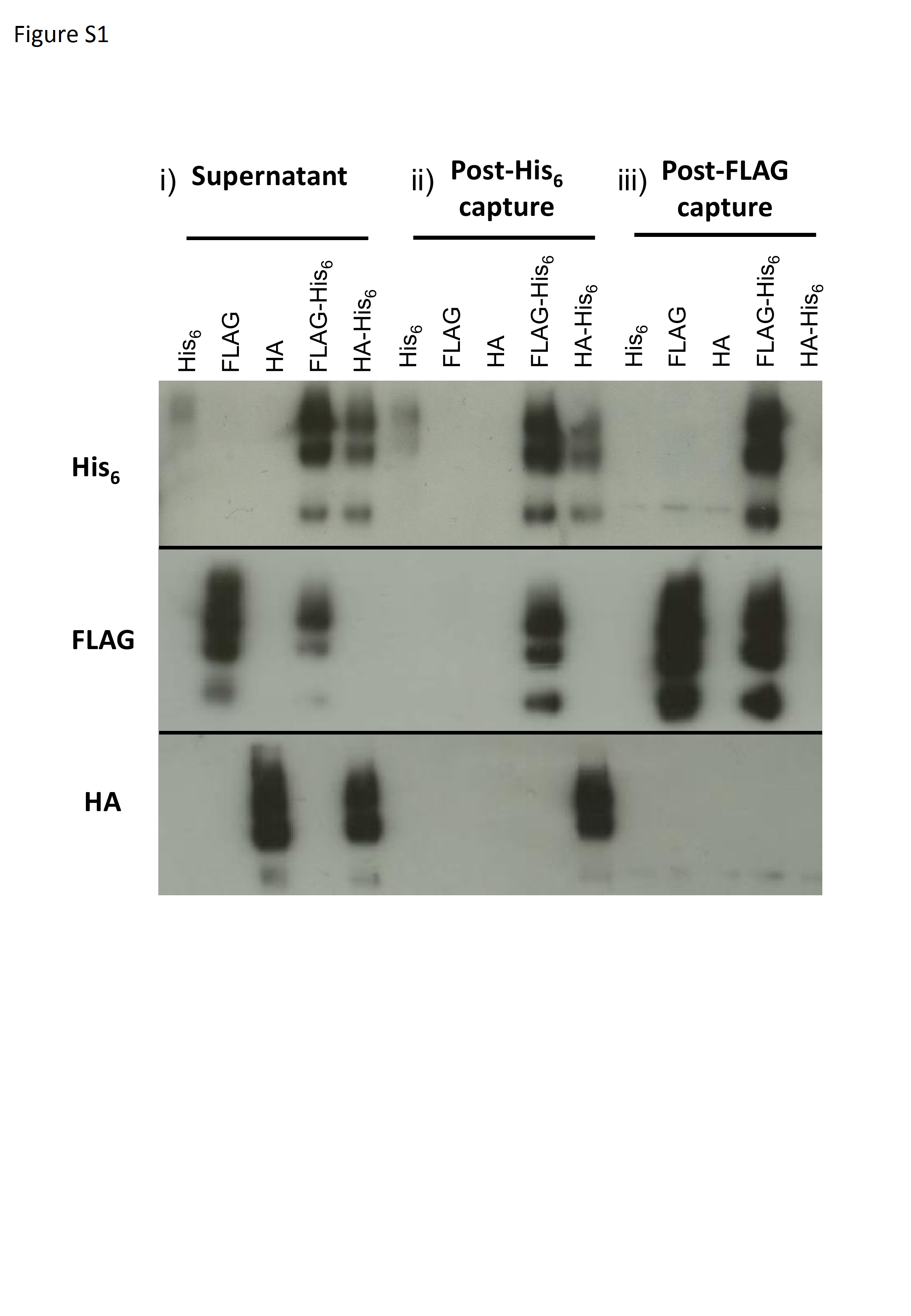

Supplement: S1 Figure — Western blot analysis to detect specific capture and detection of Env trimers with either one or two tags in series. Samples were separated by SDS-PAGE and analysed separately by western blot with anti-His6, anti-FLAG and anti-HA antibodies. Plasmids: 1 (His6), 2 (FLAG), 3 (HA), 4 (FLAG-His6) and 5 (HA-His6). (i) Rows 1–5: Supernatants following transient transfection with plasmids 1–5; (ii) Rows 6–10: Elutions from Co2+ column (specific for His6) following capture of supernatants 1–5; (iii) Rows 11–15: Elutions from anti-FLAG-tagged magnetic beads following capture of supernatants 1–5. (TIF) [file pone.0114709.s001.tif]

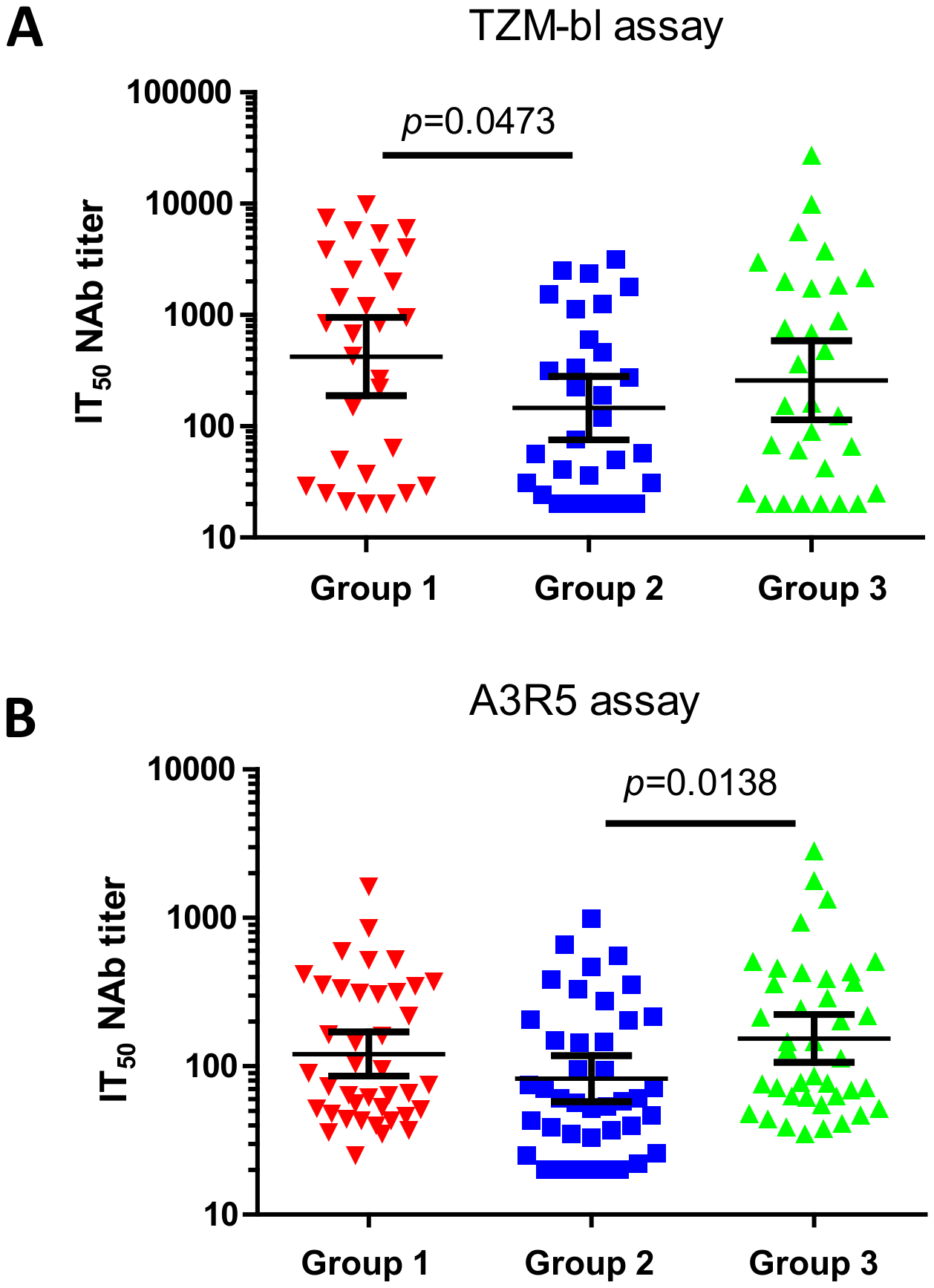

Supplement: S2 Figure — Composite analysis of neutralization data. Tat-regulated Luc reporter gene expression was determined to quantify reductions in virus infection in either (A) TZM-bl or (B) A3R5 cells. Neutralization titers are the sample dilution at which relative luminescence units (RLU) were reduced by 50% compared to RLU in virus control wells after subtraction of background RLU in cell control wells. Comparisons between groups were made individually using Mann Whitney tests. Significance at p<0.05 is indicated. (TIF) [file pone.0114709.s002.tif]

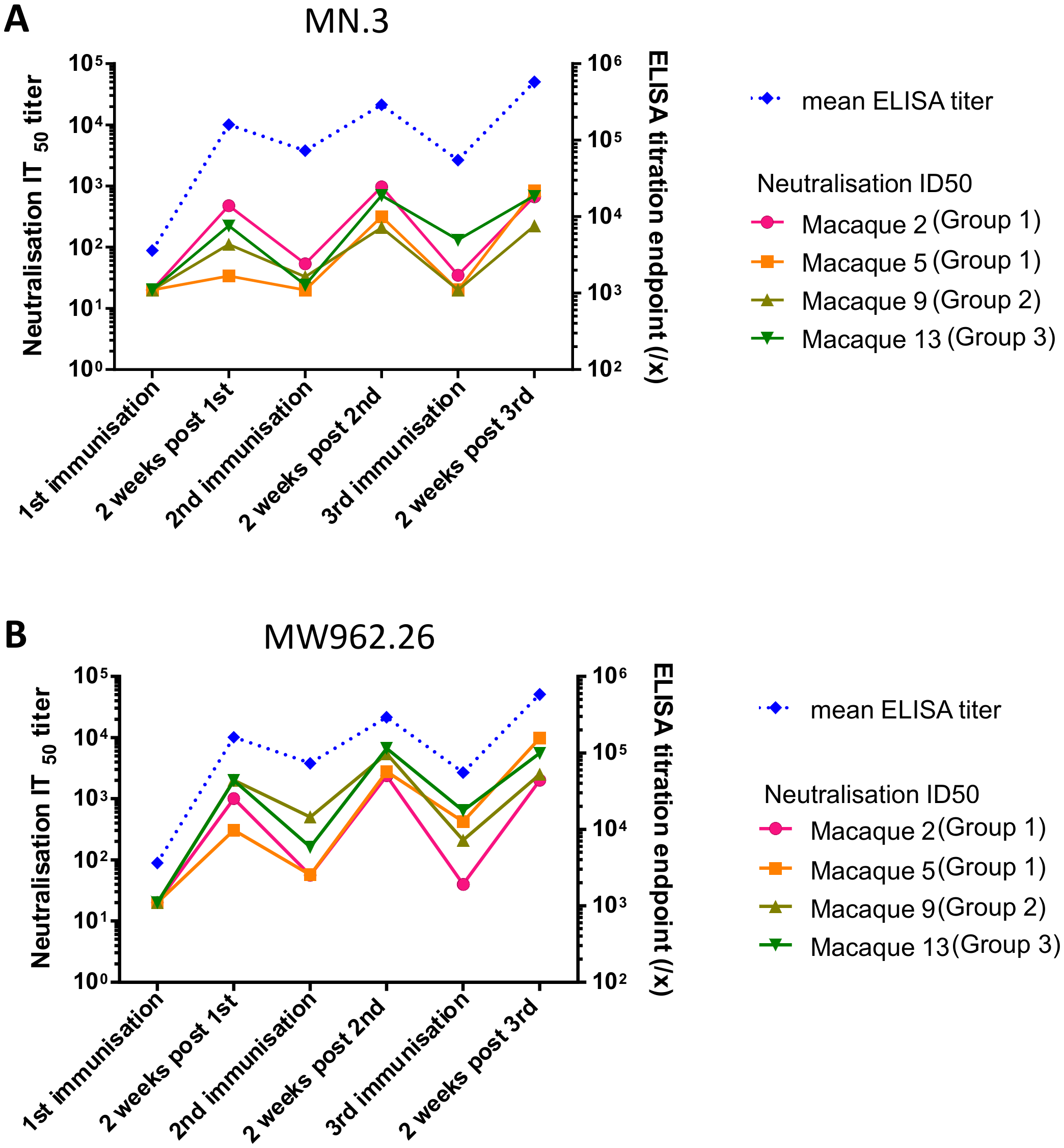

Supplement: S3 Figure — TZM-bl neutralization ID50 titers in four macaques throughout the protein immunization time-course. Macaque 2 (Group 1), Macaque 5 (Group 1), Macaque 9 (Group 2) and Macaque 13 (Group 3). (A) MN.3 (tier 1, subtype B). (B) MW965.26 (tier 1, subtype C). In both, individual neutralization titers are shown for each of the four macaques on the left-hand axis, and the mean gp140 ELISA titer of these four macaques is shown on the right-hand axis as serum dilution factors (1/x). (TIF) [file pone.0114709.s003.tif]
