# Supplementary material for: Comparison of Neutralizing Antibody Responses Elicited from Highly Diverse Polyvalent Heterotrimeric HIV-1 gp140 Cocktail Immunogens versus a Monovalent Counterpart in Rhesus Macaques
Source: PLoS One. 2014 Dec 9;9(12):e114709. doi: 10.1371/journal.pone.0114709 (PMC4260879; doi:10.1371/journal.pone.0114709)
Supplement: S1 Table — Primer details. Table of primers used for cDNA library formation from purified viral RNA extracted from human serum and amplification of env genes by RT-PCR and PCR for cloning into the expression vector pLEXm. (DOC) [file pone.0114709.s004.doc]

| **Table S1. Primer details** | | |
| --- | --- | --- |
| *RT-PCR amplification of env from viral RNA* | | |
| **EnvA (Fw)*** | GGC TTA GGC ATC TCC TAT GGC AGG AAG AA | Annealing at 65°C for 3 cycles, 60°C for 11 cycles and 55°C for 26 cycles |
| **Env3Rlong (Rev)** | GGT GTG TAG TTC TGC CAA TCA GGG AAG WAG CCT TGT GTG |
| **Env2Flong (Fw)** | GGT TAA TTG ATA GAA TWA GRG AAA GAG CAG AAG ACA GTG GCA ATG | Annealing at 55^o^C |
| **Nef5 (Rev)** | CCC WTC CAG TCC CCC CTT TTC TTT TAA AAA G |
|  | | |
| *Sequencing of gp160 env* | | |
| **M13F (Fw)** | GTAAAACGACGGCCAG |  |
| **M13R (Rev)** | CAGGAAACAGCTATGAC |  |
| **Senv5 (Fw)** | GGTACCTGTGTGGAAAGA |  |
| **F112 (Fw)** | CAGTACAATGYA CACATGGRA |  |
| **EnvSeqF (Fw)** | TTCAGACCTGGAGGAGGARATATGA |  |
| *Incorporation of tPA leader sequence, tags and restriction digest sites into pLEXm backbone vector* | | |
| **plecTPAF (Fw)** | GGCCAATTGGCCGCCACCATGGATGCAATGAAGAGAGGG | |
| **plecTPA_flagR (Rev)** | CCGATTCAGAAGAGGAGCCAGATCCGGGAATTCGGTACCCTCGAGGATTACAAGGATGACGACGATAAGTAGGTCGACGC | |
| **plecTPA_hisR (Rev)** | TGCCCGATTCAGAAGAGGAGCCAGATCCGGGAATTCGGTACCCTCGAGAAACACCATCACCACCACCACTAGGTCGACGC | |
| **plecTPA_haR (Rev)** | CATGCCCGATTCAGAAGAGGAGCCAGATCCGGGAATTCGGTACCCTCGAGTATCCTTACGACGTTCCTGACTATGCTTAGGTCGACGC | |
| **plecTPA_flaghisR (Rev)** | CATGCCCGATTCAGAAGAGGAGCCAGATCCGGGAATTCGGTACCCTCGAG GATTACAAGGATGACGACGATAAGCACCATCACCACCACCACTAGGTCGACGC | |
| **plecTPA_hahisR (Rev)** | CATGCCCGATTCAGAAGAGGAGCCAGATCCGGGAATTCGGTACCCTCGAG TATCCTTACGACGTTCCTGACTATGCTCACCATCACCACCACCACTAGGTCGACGC | |
|  |  | |
| *Amplification of env gp140 for insertion into pLEXm-TPA constructs* | | |
| **LibFEcoR1 (Fw)** | GGAATTCCGGTGGG TCACAGTCT ATTATGGGGTAC C | |
| **LibR140xho1 (Rev)** | CCGCTCGAG CCTAYYAARCCYCCTACTATYATKA | |

*All primers are shown in 5’-3’ orientation; (Fw) forward primer; (Rev) reverse primer
