# Supplementary material for: Comparison of Neutralizing Antibody Responses Elicited from Highly Diverse Polyvalent Heterotrimeric HIV-1 gp140 Cocktail Immunogens versus a Monovalent Counterpart in Rhesus Macaques
Source: PLoS One. 2014 Dec 9;9(12):e114709. doi: 10.1371/journal.pone.0114709 (PMC4260879; doi:10.1371/journal.pone.0114709)
Supplement: S2 Table — Characterisation of gp140s by tandem mass spectrometry. Table of Env peptides sequences derived from tandem mass spectrometry for either UG37 gp140 Env or heterotrimer Env samples. (DOC) [file pone.0114709.s005.doc]

| **Table S2. Characterisation of gp140s by tandem mass spectrometry** | | | | |
| --- | --- | --- | --- | --- |
| Specific gp140 identified | Protein score* | Sequence coverage [%]^†^ | Peptides (unique to specific gp140) |  |
|  |  |  |  |  |
| UG37 | 4546 | 62 | AIEAQQHLLK |  |
|  |  |  | NEKDLLELDK |  |
|  |  |  | TLHQVVEQLRK |  |
|  |  |  | CESNITGLILTR |  |
|  |  |  | QAHCNVSGSQWNK |  |
|  |  |  | CNDKEFNGTGLCK |  |
|  |  |  | MENVTEEFNMWK |  |
|  |  |  | LLSGIVQQQSNLLR |  |
|  |  |  | VGQAMYAPPIQGVIK |  |
|  |  |  | MENVTEEFNMWK |  |
|  |  |  | VGQAMYAPPIQGVIK |  |
|  |  |  | LINCNTSALTQARPK |  |
|  |  |  | IYELIEESQIQQER |  |
|  |  |  | EEIKNCSFNMTTELR |  |
|  |  |  | IGPGQTFYATGDIIGDIR |  |
|  |  |  | LDVVQINNGNNSSNLYR |  |
|  |  |  | LDVVQINNGNNSSNLYR |  |
|  |  |  | DGGVNSSDSETFRPGGGDMR |  |
|  |  |  | SLDEIWENMTWLQWDK |  |
|  |  |  | VTFEPIPIHYCAPAGYAILK |  |
|  |  |  | NIIVQLNESVTINCTRPNNNTR |  |
|  |  |  | AYDTEVHNVWATHACVPTDPSPQELK |  |
|  |  |  | NVSTVQCTHGIRPVVSTQLLLNGSLAEGK |  |
|  |  |  |  |  |
| NIB_52_4 (Mix 1)^#^ | 932 | 23 | QAYCNVSR |  |
|  |  |  | EEWNDALR |  |
|  |  |  | QIMNMWQR |  |
|  |  |  | NCSYNMTTELR |  |
|  |  |  | DIISLWDQSLKPCVK |  |
|  |  |  | GIHIGPGQAFYATDNIIGNIR |  |
|  |  |  | VGQAMYAPPIQGVITCESNITGLLLTR |  |
|  |  |  |  |  |
| NIB_34_6 (Mix 3) | 896 | 25 | AFYAMGR |  |
|  |  |  | KLGEQFGNK |  |
|  |  |  | EEGEIKGEIK |  |
|  |  |  | QVINMWQEVGK |  |
|  |  |  | NSSREEGEIKGEIK |  |
|  |  |  | DHVEINCTRPNNNTSK |  |
|  |  |  | DGGNETNTTEVFRPGGGDMR |  |
|  |  |  |  |  |
| NIB_1_3 / 1_4 (Mix 3) | 695 | 21 | KSIPIGPGR |  |
|  |  |  | TFYTTGEIIGDIR |  |
|  |  |  | SVEINCTRPNNNTR |  |
|  |  |  | EYALFYELDIIPIK |  |
|  |  |  | EIDNYTDSIYTLIEK |  |
|  |  |  |  |  |
| NIB_83_4/83_5 (Mix 1) | 533 | 17 | IEPLGVAPTHAK |  |
|  |  |  | TIIVQLNKPVR |  |
|  |  |  | GIGIGPGQMFYAADAIIGDIR |  |
|  |  |  | KGIGIGPGQMFYAADAIIGDIR |  |
|  |  |  |  |  |
| NIB_21_5/42_2/8_2 (Mix 1) | 475 | 11 | VEPLGVAPTR |  |
|  |  |  | IRSENITNNAK |  |
|  |  |  |  |  |
| NIB_41_1 (Mix 1) | 299 | 14 | VGQAMYAPPIAGVIK |  |
|  |  |  | TVQINCTRPNNNTR |  |
|  |  |  |  |  |
| NIB_44_6 (Mix 1) | 347 | 13 | AIEAQQHLLR |  |
|  |  |  | VGQAMYAPPIR |  |
|  |  |  |  |  |
| NIB_9_3/9_6 (Mix 4) | 324 | 8 | SNITGILLTR |  |
|  |  |  | LINCNTSAVTQACPK |  |
|  |  |  | TPVEIVCTRPGNNTR |  |
|  |  |  |  |  |
| NIB_38_6/52_4 (Mix 1) | 268 | 11 | LLEDSQNQQEK |  |
|  |  |  |  |  |
| NIB_14_10 (Mix 2) | 257 | 9 | AMYAPPIQGLISCSSNITGLLLTR |  |
|  |  |  | AMYAPPIQGLISCSSNITGLLLTR |  |
|  |  |  |  |  |
| NIB_67_3 (Mix 1) | 224 | 5 | LDVVQTDDSNSYR |  |
|  |  |  |  |  |
| NIB_11_6 (Mix 4) | 205 | 6 | SNITGLLLER |  |
|  |  |  |  |  |
| NIB_30_11 (Mix 5) | 172 | 6 | VVKIEPLGIAPTK |  |
|  |  |  |  |  |
| NIB_73_9 (Mix 4) | 95 | 4 | QIINLWQGVGR |  |
|  |  |  | LLEESQNQQER |  |

*Protein score: Mascot protein scores represent the –10 log value of the probability of this protein match. Peptides selected based on uniqueness to this gp140 subtype and a Mascot peptides score >20.

^#^NIB numbers are unique identifiers that allow designation of individual Env peptide sequences to Mixes 1-5 that were detected as being present in Mix 6 gp140 protein.

^†^Sequence coverage is the % of the total protein sequence of the reference full-length Env sequence which has been identified by the tandem mass spectrometry sequencing experiment.
