# Supplementary material for: Comparison of Neutralizing Antibody Responses Elicited from Highly Diverse Polyvalent Heterotrimeric HIV-1 gp140 Cocktail Immunogens versus a Monovalent Counterpart in Rhesus Macaques
Source: PLoS One. 2014 Dec 9;9(12):e114709. doi: 10.1371/journal.pone.0114709 (PMC4260879; doi:10.1371/journal.pone.0114709)
Supplement: S3 Table — ELISA titration end-points. Table of ELISA end point titers for individual macaque sera at all experimental sampling timepoints. (DOCX) [file pone.0114709.s006.docx]

**Table S3. ELISA titration end-points**

| **Test subject** | **Pre-immunisation** | **First immunisation** | **2 weeks post immunisation** | **2nd immunisation** | **2 weeks post 2nd immunisation** | **3rd immunisation** | **2 weeks post 3rd immunisation** | **Final bleed** |
| --- | --- | --- | --- | --- | --- | --- | --- | --- |
| **Group 1** |  |  |  |  |  |  |  |  |
| 1 | 3.80E+03^#^ | 2.75E+03 | 5.18E+04 | 4.19E+03 | 2.63E+05 | 1.99E+04 | 2.01E+05 | 4.90E+04 |
| 2 | 7.68E+02 | 4.23E+02 | 1.18E+05 | 7.76E+03 | 1.39E+05 | 6.09E+03 | 1.28E+05 | 3.96E+04 |
| 3 | 1.67E+03 | 1.58E+03 | 1.10E+05 | 7.94E+03 | 2.99E+05 | 1.29E+04 | 3.00E+05 | 5.22E+04 |
| 4 | 5.28E+02 | 1.24E+03 | 1.01E+05 | 9.96E+03 | 2.45E+05 | 2.49E+04 | 2.72E+05 | 1.87E+05 |
| 5 | 1.14E+03 | 1.03E+03 | 7.33E+04 | 4.24E+03 | 1.93E+05 | 2.54E+04 | 2.85E+05 | 6.34E+04 |
| ***Average*** | ***1.58E+03*** | ***1.41E+03*** | ***9.09E+04*** | ***6.82E+03*** | ***2.28E+05*** | ***1.78E+04*** | ***2.37E+05*** | ***7.83E+04*** |
| **Group 2** |  |  |  |  |  |  |  |  |
| 6 | 8.83E+03 | 2.54E+03 | 1.17E+05 | 1.15E+05 | 3.33E+05 | 2.20E+04 | 2.32E+05 | 3.50E+04 |
| 7 | 1.12E+03 | 8.82E+02 | 5.48E+04 | 4.63E+04 | 2.51E+05 | 3.29E+04 | 1.69E+05 | 4.55E+04 |
| 8 | 1.29E+03 | 1.23E+03 | 5.27E+04 | 1.09E+05 | 4.62E+05 | 1.55E+04 | 2.05E+05 | 2.79E+04 |
| 9 | 3.84E+03 | 1.14E+04 | 1.37E+05 | 6.72E+04 | 3.77E+05 | 9.45E+04 | 9.67E+05 | 1.09E+05 |
| 10 | 1.99E+04 | 3.40E+02 | 4.06E+04 | 2.94E+04 | 1.51E+05 | 4.30E+04 | 4.12E+05 | 8.85E+04 |
| ***Average*** | ***6.99E+03*** | ***3.28E+03*** | ***8.04E+04*** | ***7.34E+04*** | ***3.15E+05*** | ***4.16E+04*** | ***3.97E+05*** | ***6.11E+04*** |
| **Group 3** |  |  |  |  |  |  |  |  |
| 11 | 2.70E+03 | 1.69E+03 | 1.62E+05 | 2.99E+04 | 3.49E+05 | 1.39E+04 | 1.58E+05 | 3.55E+04 |
| 12 | 2.36E+04 | 5.02E+02 | 2.12E+05 | 4.31E+04 | 1.10E+06 | 1.37E+04 | 3.94E+05 | 9.41E+04 |
| 13 | 1.05E+04 | 1.68E+03 | 2.32E+05 | 8.83E+04 | 2.23E+05 | 2.26E+04 | 2.10E+05 | 7.44E+04 |
| 14 | 4.47E+03 | 1.64E+03 | 3.11E+05 | 2.14E+05 | 4.54E+05 | 9.48E+04 | 9.52E+05 | 1.58E+05 |
| 15 | 1.38E+03 | 9.06E+02 | 2.11E+05 | 2.70E+04 | 5.00E+05 | 4.23E+04 | 2.50E+05 | 4.69E+04 |
| ***Average*** | ***8.54E+03*** | ***1.28E+03*** | ***2.26E+05*** | ***8.05E+04*** | ***5.26E+05*** | ***3.75E+04*** | ***3.93E+05*** | ***8.18E+04*** |
|  |  |  |  |  |  |  |  |  |

^#^All sera were tested against a plating antigen of UG37 gp140. Numbers given in the table are reciprocal serum dilutions of end-point titers
